# The Effects and Potential Mechanism of Oil Palm Phenolics in Cardiovascular Health: A Review on Current Evidence

**DOI:** 10.3390/nu12072055

**Published:** 2020-07-10

**Authors:** Nurul ‘Izzah Ibrahim, Syed Fairus, Isa Naina Mohamed

**Affiliations:** 1 Pharmacoepidemiology and Drug Safety Unit, Department of Pharmacology, Faculty of Medicine, Universiti Kebangsaan Malaysia Medical Centre, Kuala Lumpur 56000, Malaysia; nurulizzah88@gmail.com; 2 Malaysian Palm Oil Board (MPOB), No. 6 Persiaran Institusi, Bandar Baru Bangi, Kajang Selangor 43000, Malaysia; syfairus@mpob.gov.my

**Keywords:** hyperlipidemia, oil palm phenolics, cholesterol biosynthesis pathway

## Abstract

Cardiovascular disease (CVD) is globally known as the number one cause of death with hyperlipidemia as a strong risk factor for CVD. The initiation of drug treatment will be recommended if lifestyle modification fails. However, medicines currently used for improving cholesterol and low-density lipoprotein cholesterols (LDL-C) levels have been associated with various side effects. Thus, alternative treatment with fewer or no side effects needs to be explored. A potential agent, oil palm phenolics (OPP) recovered from the aqueous waste of oil palm milling process contains numerous water-soluble phenolic compounds. It has been postulated that OPP has shown cardioprotective effects via several mechanisms such as cholesterol biosynthesis pathway, antioxidant and anti-inflammatory properties. This review aims to summarize the current evidence explicating the actions of OPP in cardiovascular health and the mechanisms that maybe involved for the cardioprotective effects.

## 1. Introduction

Cardiovascular disease (CVD) is globally known as the number one cause of death with increasing numbers of people dying annually due to CVD compared to other diseases. An estimation of 18 million death per year from CVD has been made, which represents 31% of all death caused by disease globally [1]. CVD can be categorized as the heart and blood vessel diseases including coronary heart disease, cerebrovascular disease and other conditions. Individuals who are at risk of CVD may show significant increment of glucose and lipid levels in the blood, raised blood pressure and significant changes in body mass index (BMI) [2]. 

Hyperlipidemia, which has been well established as a potent risk factor for CVD, can be defined as an isolated elevation of fasting total cholesterol (TC) concentration, which may or may not be related to isolated elevation of triglyceride (TG) concentration [3]. A strong link has been identified between elevated cholesterol especially low-density lipoprotein cholesterols (LDL-C) and CVD [4]. According to the guidelines by the National Cholesterol Education Program (NCEP), lifestyle modification (diet and exercise) should be performed before initiation of drug treatment [5]. Lowering of LDL-C in patients with intermediate to high risk for CVD has also been a well-accepted medical practice combined with lifestyle changes [3]. Statins, the first-line drug therapy, are recommended for initial treatment at a low dose to prevent adverse events and then titrated to keep LDL-C at or below the recommended target range (<100 mg/dL) [3,6]. However, statins have been associated with serious adverse reactions including muscle damage, renal failure, liver dysfunction and polyneuropathy [7]. Moreover, the controversial issue of whether or not statins could cause type 2 diabetes has been recently raised [8,9]. Multiple alternatives are also available such as ezetimibe, fibrates and niacin for patients who are statin-intolerant or fail to attain therapeutic goals. Unfortunately, these alternatives have also been associated with various adverse reactions namely ezetimibe (diarrhea, arthralgia, nasopharyngitis, controversial regarding CVD events reduction), fibrates (dyspepsia, gallstones, myopathy) and nitrates (hyperglycemia, hyperuricemia and hepatoxicity) [3,10]. Medicines currently used for improving cholesterol and LDL-C levels have their own side effects, suggesting a dire need to search for alternative therapies with fewer or no side effects. Natural products containing phenolic compounds have demonstrated potential cardiovascular protective effects with promising safety and were thus considered as alternative treatments [11,12,13,14,15,16]. For example, *Primula veris* L. extract that contains various phenolics such as aglycons and glycosides has demonstrated increased myocardial contractility with a good safety profile in a heart failure experimental study [13]. Additionally, several natural products of Traditional Chinese Medicine such as NeuroAid/MLC 901 and Xin-ke-Shu have been used in China for reducing and treating cerebral infarction [14] and coronary heart disease [16], respectively. These studies suggested that natural products have been accepted and considered as alternative treatments. Therefore, other natural products should be explored to obtain more alternative treatments for cardiovascular diseases.

The cultivation of an agricultural plant known as African oil palm (*Elaeis guineensis* Jacq.) is mainly done for palm oil production [17]. Generally, palm oil is cultivated mainly in Asia, Africa and Latin America. The top palm oil producers are Indonesia and Malaysia, followed by Thailand, Colombia and Nigeria [18]. Palm oil is the world’s top producing and cheapest vegetable oil, making it favored by millions of people for its role as edible oil [17,19]. In addition, palm oil is known for its traditional use as an antidote for many illnesses such as gastrointestinal disorders and poisons [20]. In production industries, palm oil and its derivatives are utilized for manufacturing processed foods, personal care and beauty products as well as household cleaning products [17]. Due to the high demand in the manufactured products, the production volume of palm oil is estimated to grow by 3 million metric tons from the marketing year 2017/2018 to 2018/2019 [18].

In the oil palm industry, palm oil only covers up to 10% while the remaining 90% consist of solid, aqueous and refined by-products [21,22]. During the milling process, disposal of enormous amounts of aqueous by-products in the waste stream contributes to 85 million tons of waste per year worldwide [22,23]. Fine solids separated from the aqueous waste were used as animal feed or fertilizers [22]. Only recently, innovations to retrieve phenolic compounds from the low-value and high-volume aqueous stream have been developed, resulting in a filtrate production known as oil palm phenolics (OPP) [24]. Although OPP is originated from oil palm, which is a common plantation in certain regions, its traditional use is still limited since OPP is a by-product generated from the milling process. Moreover, research and development in discovering the OPP beneficial effect is yet to be investigated. 

Driven by the increasing evidence that phenolics originated from plants are valuable, the OPP was carefully evaluated for its positive bioactivities that have been found to possess health beneficial properties [25]. The high amount of phenolics in OPP has provided a chance to convert palm milling waste into an array of health and wellness potential use [23]. It has been postulated that the water-soluble phenolics found in OPP have beneficial medicinal properties such as antitumor [26], neuroprotective [27] and cardioprotective effects [28]. Several studies have revealed that the water-soluble phenolic compounds present in other natural products may exhibit cardioprotective properties. In a study by Katengua-Thamahane et al. (2014), rooibos, which is a South African herbal tea that contains high levels of polyphenols, exerted cardioprotective effects on the myocardial tissue via its anti-inflammatory property [29]. Phenolics extracted from grapes also prevented the initial development of atherosclerotic lesions in atherogenic diet-fed hamsters [30]. 

**Figure 1:**
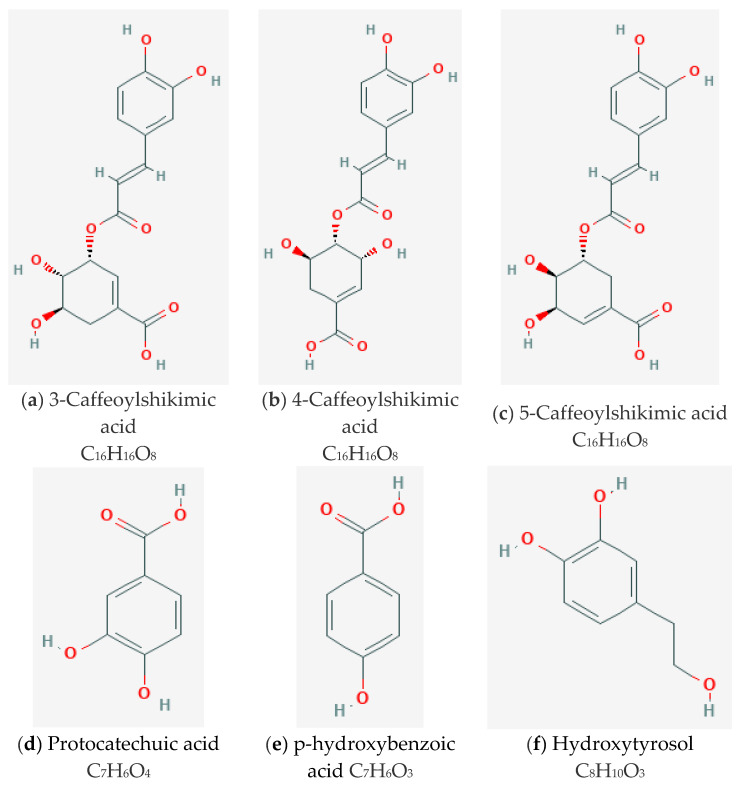
The chemical structures of main components of Oil Palm Phenolics; (**a**) 3-Caffeoylshikimic acid; (**b**) 4-Caffeoylshikimic acid (**c**) 5-Caffeoylshikimic acid; (**d**) Protocatechuic acid (PCA); (**e**) p-hydroxybenzoic acid (PHBA); (**f**) Hydroxytyrosol (HT). 3-Caffeoylshikimic acid, 4-Caffeoylshikimic acid and 5-Caffeoylshikimic acid are the isomers of caffeoylshikimic acid.

OPP contains numerous water-soluble phenolic compounds such as caffeoylshikimic acid (CFA) as the main component and other phenolic acids including caffeic acid, protocatechuic acid (PCA), as well as p-hydroxybenzoic acid (PHBA) and hydroxytyrosol (HT) [23]. Their chemical structures are depicted in Figure 1. These natural hydrophilic phenols are associated with antioxidant capacity that can suppress reactive oxygen species (ROS) and potentially curb the development of pathogenic mechanisms related to CVD [31]. Besides that, OPP has been proposed in preventing and treating CVD via several mechanisms including modulation of biochemistry and metabolism pathways [32]. In addition, the effects of OPP as a cardioprotective agent have been also associated with its potential anti-inflammatory property [27]. 

This review elucidates the effects of OPP and its postulated cardioprotective mechanism in preventing and treating CVD. This information will be beneficial and relevant for future research on CVD and the development of a comprehensive natural medicine approach to CVD. 

## 2. The Effects of OPP in Cardiovascular Health

The direct effects of OPP in cardiovascular health have been previously investigated, as tabulated in Table 1. In these studies, OPP has demonstrated its effects in atherosclerosis, cardiac arrhythmia and hypertension. For instance, Che Idris et al. (2014) explored the effects of OPP against atherosclerosis in an atherogenic rabbit model. It was described that the OPP-treated rabbits had significantly less fibrous plaques compared to the atherogenic diet control rabbits, suggesting that OPP might inhibit atherosclerotic lesion development. The OPP-treated rabbits also displayed proliferation of smooth muscles, presence of intimal fibrosis and extracellular lipid, but absence of lipid core or necrosis. Additionally, the formation of foam cell with lesser degree has been also reported. However, in this study, the plasma lipid profile of OPP-treated group was insignificant compared to the atherogenic diet control group [28]. 

Abeywardena et al. (2014) [33] conducted a set of animal experimentations using different rat models for acute and chronic administration of OPP to evaluate the potential cardiovascular outcomes. In their experiment, different models of hypertensive rat models were used, namely as spontaneously hypertensive rats (SHR) and L-NAME-induced hypertensive rats. They found that the chronic (20 weeks) OPP administration (1500 mg/L GAE) showed no effect on the blood pressure of SHR rats, which were genetically modified hypertension models. In contrast, the administration of OPP for three months had significantly lowered the blood pressure in the L-NAME-induced hypertension rat models. However, the lowering effect did not cause a complete normalization of blood pressure to the baseline level of the control rats [33]. The prolonged use of L-NAME to induce hypertension is a well-established experimental model that can be characterized by generalized NO deficiency, causing a progressive increase in BP [35]. Additionally, Abeywardena and his team [33] also reported that at a higher OPP dose (3000 mg/L GAE), significant cardioprotective effects were observed in the L-NAME-induced rats. This was demonstrated by the significantly reduced ventricular tachycardia duration where OPP had 2.3 s, whereas the control had 35.7 s. Plus, ventricular fibrillation episodes were absent in the OPP-treated group. In an acute administration of fractionated OPP to the spontaneously hypertensive rats (SHR), a considerable reduction in mean BP (7–27 mmHg) has been shown within a 6-h window following the administration. The highest reduction mediated by both OPP fractions (250 mg/kg each) was at 6 h post-administration. The effect of OPP was compared to an anti-hypertensive agent, enalapril (4 mg/kg), which peaked at 9 h following administration [33]. This suggests that OPP had a much shorter duration to reach peak concentration compared to enalapril, indicating the potential of OPP to rapidly reduce BP. 

Abeywardena et al. (2014) [33] also performed an experiment to observe the effects of OPP in cardiac arrhythmia-induced rats via coronary artery ligation. In comparison to control group, the OPP provided as a beverage had a significant reduction of incidence percentage (*p* < 0.05). For ventricular fibrillation (VF) incidence, the OPP-treated group had a lower rate of 52% compared to the control group with 90% incidence. The supplementation of OPP also demonstrated a lower mortality rate of 20% in comparison with a control group of 40% [33]. The reduction in vulnerability to cardiac arrhythmias implicates the effectiveness of OPP as an active agent in lowering CVD mortality and morbidity. 

The potential of OPP fractions in lowering vascular resistance has been tested using noradrenaline pre-contracted aortic ring and mesenteric vascular assays [23]. This model has been used to investigate the potential vasodilatory effects induced by endothelial-derived nitric oxide (NO), which may trigger relaxation and dilation to the smooth muscle. It was evident that OPP fractions had dose-dependently enhanced vascular relaxation in both ex vivo assays. These results indicate that the ability of OPP to cause vascular relaxation is facilitated by endothelial NO, which subsequently initiates relaxation and dilatation of the smooth muscle. The promoted vascular relaxation in the ex vivo preparations may suggest that OPP could be an effective agent in lowering the BP in the whole animal. In the same paper, the authors also investigated the inhibition of copper (Cu)-mediated LDL oxidation by OPP, which demonstrated an inhibition of the in vitro human LDL oxidation-mediated copper in a dose-dependent manner. Several in vitro studies have revealed the ability of phenolics in preventing LDL oxidation via free radical scavenging and metal ion sequestration [36,37,38]. The inhibition of LDL oxidation by OPP was dose dependent as reflected by the prolonged duration of conjugated diene formation with increasing OPP concentration. This implies the potential of OPP to prevent fatty deposition in the arteries. Apart from that, the binding of plant polyphenols has been associated with cardiovascular benefits in relation to LDL oxidation. Various plant phenols such as quercetin, resveratrol, PCA and PHBA have showed their ability to relatively bind with LDL [39]. It has been reported that the binding of LDL by plant phenols showed better resistance to oxidation than LDL alone [40]. Since OPP contains various plant phenols including PCA and PHBA, this may contribute to its cardioprotective effects. The oxidative modification of LDL has been identified to contribute to the pathogenesis of atherosclerosis. Oxidized LDL is taken up by macrophages and smooth muscle cells, subsequently initiating the formation of fatty streaks, an initial event during atherosclerosis progression [41,42]. Thus, the protection against LDL oxidation by OPP may indicate a reduction in CVD risk. 

In a molecular study using microarray gene expression profiling on mice fed a 6-week normal diet, Leow et al. [32] reported that OPP showed positive outcomes on cardiovascular effects in mice liver. In the study, OPP had upregulated four lipid catabolism genes including Acadl, Acads Hadhb and Hadhsc, while it downregulated five cholesterol biosynthesis genes namely Hmgcs1, Lss, Sc4mol, Fdps and Nsdhl in the liver. The changes were classified as low fold, which suggest that OPP does not generally modulate gene expression drastically and is potentially suitable to be developed as dietary supplements. Supplement intake should avoid overwhelming effects on the body systems. It is recommended for a supplement to be able to buffer the effect of oxidative stress via antioxidant action. This gene modulation might demonstrate the potential of OPP to reduce CVD risk. In a different molecular study using microarray gene expression profiling on atherogenic diet-fed mice, Leow et al. (2013) [27] discovered that OPP has caused attenuated atherogenic oxidative stress, inflammation and the high turnover of cells and metabolites in liver and heart of the mice. In addition, OPP had significantly increased serum HDL level [27]. 

The positive results of OPP related to CVD in the preclinical studies further suggested its possible application in humans. A phase 1 clinical trial has been performed in healthy subjects to assess the effects of OPP on physiologic condition. The subjects were supplemented with OPP at 450 mg GAE/day for 60 days, which eventually demonstrated significantly lower plasma TC (*p* = 0.025) and LDL-C (*p* = 0.04) compared to the control treatment. However, the significant result was only obtained when using the Wilcoxon signed rank test. Following Bonferroni correction, the reduction in TC and LDL-C was insignificant. In addition, the supplementation of OPP showed insignificant levels of triglyceride (TG) and high-density lipoprotein cholesterol (HDL-C) when compared to control subjects. Nevertheless, it should be noted that the subjects were healthy with normal range of lipid profiles [34]. Therefore, any insignificant changes following the OPP supplementation were anticipated. In most cases, a lack of significant change was reported in healthy subjects when phenolic substances were supplemented [43,44,45,46]. Both previously human intervention trial with red wine [44] and cranberry juice [45] for two weeks duration did not significantly change the lipid profiles in the plasma of healthy subjects. In another study, the consumption of pomegranate polyphenol extract for four weeks in a healthy population also did not reveal significant improvement in plasma TAG, TC, HDL-C and LDL-C levels [46]. In accordance with the positive outcomes from the existing preclinical studies [43,44,45,46], OPP may offer a promising effect to improve the lipid profiles in subjects with elevated levels of TC. 

As reported in a recent review [47], each individual component of OPP namely PCA, PHBA, CFA and hydroxytyrosol possess unique pharmacological potential including cardiovascular preventive related properties such as anti-atherosclerosis, cardioprotection and hypolipidemic effects. PCA, which is a derivative of PHBA, is commonly occurring in various plants including olives [48], grapes [49] and roselle [50]. PCA has demonstrated a triglyceride-lowering effect in plasma, heart as well as liver in diabetic-induced mice [51] and hyperlipidemic-induced rats [52], besides an anti-atherosclerosis effect as evidenced by the reduction of VCAM-1 and ICAM-1 in the plasma of apolipoprotein E-deficient mice [53]. Hydroxytyrosol (HT), is commonly detected in the natural plants including palm oil [54] and olives [55]. In a review by Borzi et al. (2018), it was seen that HT from olive oil possessed chemotherapeutic potential against colorectal cancer [56], indicating the protective role of HT in diseases other than CVD. In a review by Visioli et al. (2019), HT that is also present in extra virgin olive oil has been described to possess pharmacological prominent effects concerning cardiovascular system [57]. HT exhibited cardioprotective effects through the reduction of TG and TC levels and increased HDL-C in hyperlipidemic-induced rabbits at the same time. Furthermore, the size of atherosclerotic lesion was reduced [58]. In a controlled randomized trial, HT exhibited the ability to improve the lipid profile and reduce lipid oxidative damage [59]. The anti-atherosclerotic effect of HT has also been confirmed in an in vitro study where lipopolysaccharide-stimulated VCAM-1 expression was reduced in human vascular endothelial cells. The reduction was associated with the mRNA inhibition, which reduced the attachment of monocyte cell onto endothelial cells [60]. This action has led to the inactivation of endothelial cells, an initial step in the development of atherogenesis [60,61]. All of these studies postulated that the individual components in OPP may provide beneficial effect against CVD. Hypothetically, these individual components may contribute to the enhancement of synergistic effect when consumed together in the OPP.

## 3. Mechanism of Actions of OPP in Cardiovascular Health 

### 3.1. Cholesterol Biosynthesis

**Figure 2:**
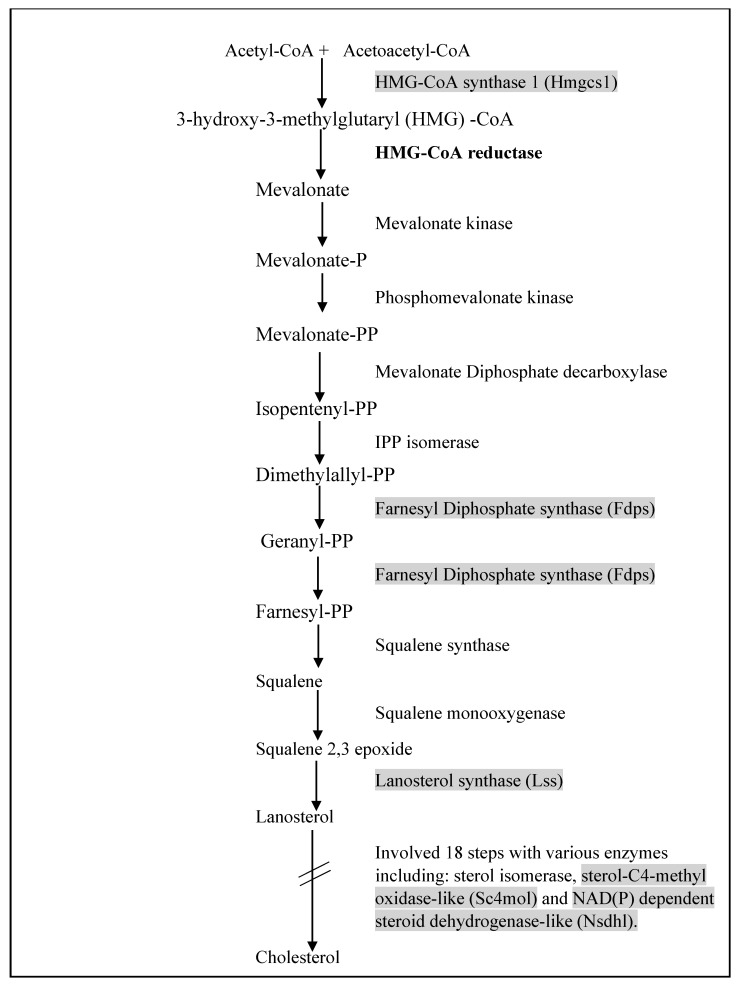
Localization of enzymes involved in the cholesterol biosynthesis pathway. In a previous study by Leow et al. (2011) [32], OPP has downregulated the highlighted genes, while the bold gene exhibited a negative fold change. The figure is modified from Sharpe et al. 2013 [64].

Cholesterol plays an important function in our body as building blocks of plasma membranes and serves as a basis for the formation of bile acid and steroid hormone. Generally, all cholesterols in the human body come from two distinct sources, which are; (i) de novo biosynthesis from acetyl-CoA by the multi-enzyme pathway or (ii) certain types of food ingestion via extracellular transport by low LDL-mediated endocytosis [62,63]. Cholesterol biosynthesis is a complex process that requires numerous enzymes and has several regulation points throughout the process. Several intermediaries involved in the biosynthesis process can be diverted and utilized as precursors for the biosynthesis of other compounds or perform specific functions in the body [62]. Leow et al. (2011) revealed that the downregulation of cholesterol biosynthesis genes by OPP in BALB/c mice might exert a hypocholesterolemic effect [32]. In their study, OPP supplemented as a beverage to the mice had downregulated genes including Hmgcs1, Lss, Sc4mol, Fdps, Nsdhl. Additionally, the OPP supplementation showed negative fold change in the expression of HMGCR gene, a statin-targeted gene responsible for lowering cholesterol level [64]. The localization of cholesterol biosynthesis genes is shown in Figure 2. Previous study has reported decreased mRNA levels of fdps and HMGR in the mouse liver fed with PUFA-rich tuna fish oil [65]. It is well known that fish oils can exert cholesterol lowering effects in rodents [66,67]. Other than that, an in vitro study using short chain fatty acids (SCFA) on human enterocyte cell lines showed downregulation of nine cholesterol biosynthesis genes including sc4mol and fdps genes, which possibly curb the pathway [68]. 

### 3.2. Antioxidant Property 

In a physiological state, reactive oxygen species (ROS) may act as carriers in signaling pathways. During homeostatic balance, fluctuation of ROS concentrations occurs under a controlled environment with the presence of antioxidants and enzymes. However, perturbation to the balanced state of ROS may cause oxidative stress to become apparent [69]. High ROS quantities may promote inflammation, lipid peroxidation as well as damaged DNA and proteins. Due to these detrimental effects, overwhelming ROS levels have been associated with pathologic conditions including CVD [70]. For CVD specifically in atherosclerosis development, the oxidative stress to LDL may generate modified particles known as oxidized particles (ox-LDL), which may enhance atherogenesis. The particles also contain the products of lipid oxidation and damaged apoprotein [71]. The presence of ox-LDL has been hypothesized to affect each stage of atherosclerosis, which consists of inflammation phase activation, endothelial damage and macrophage involvement for unregulated ox-LDL uptake to form foam cells. The foam cells, which are also known as fat-laden macrophages, are the hallmark of early atherosclerotic lesions [72]. The foam cells in progressive stages of development may burst into fatty streaks, which will mature into fatty plaques and ultimately accumulate in the arterial wall. The accumulation of fatty plaques may reduce the size of blood vessel lumen, inhibiting blood flow to the heart and brain, thus causing a heart attack or stroke [73,74]. 

The feasible connection between atherosclerosis and oxidative stress might suggest that nutritional antioxidants may inhibit atherosclerosis development [75,76]. Moreover, natural antioxidants such as vitamins (vitamin C and E), carotenoids and flavonoids have been hypothesized to interfere with atherosclerosis and CVD development by modulating the oxidation and reduction in disease progression [77,78]. In a study by Che Idris et al., OPP supplementation to atherogenic-diet fed rabbits had a significant reduction of fatty streaks and plaques [28]. Nevertheless, in comparison to the control group, the plasma antioxidant status (ABTS+ and FRAP assays) showed no significant differences. Although insignificant, the plasma antioxidant capacity (FRAP assay) of OPP-supplemented rabbits had a superior effect compared to the control group. In a previous study conducted to determine the antioxidant potential of OPP using DPPH assay, OPP has shown significant scavenging activity with a half-life (t1/2) of shorter than 30 s for the tested concentrations ranging from 100 to 300 mg/L GAE [23]. At 100 mg/L GAE, there were more than 75% DPPH assays scavenged by OPP. However, a direct antioxidant activity during in vitro and in vivo studies should be supported by molecular studies to provide evidence for the pharmacological activity at receptor, cell signaling and gene expression involved in the antioxidant activity. This is because polyphenols are poorly absorbed and rapidly degraded, which may result in very low bioavailability [79].

It has been reported that gut microbiota, which are the microorganisms living in the gut can improve the bioavailability of polyphenols. Following oral ingestion of polyphenols, the molecules must be absorbed into the body and carried through the bloodstream from the absorption site to target tissues and organs. The gut microbiota may be involved in the metabolism of polyphenols by converting dietary polyphenols to low-molecular-weight phenolic compounds (such as phenolic acids), which are more readily absorbed by intestinal epithelial cells [80]. In addition, bacterial populations in the gastrointestinal tract may also contribute to enzymatic processes of the ingested polyphenol compounds [80,81]. Polyphenols may also exhibit beneficial effects by acting as prebiotics via their selective antimicrobial activity against pathogenic bacteria on the intestinal microbiota [82]. Therefore, the gut microbiota that reside mostly in the large intestine can improve the bioavailability of polyphenols to exert beneficial effects including antioxidant property. In providing support to the in vitro and in vivo studies, a molecular study has been performed to explain the gene expression involved in the antioxidant activity of OPP. Following microarray studies in the hearts of mice, OPP showed upregulation of antioxidant gene expression such as Mgst1, Gpx1 [27], Gstm2, Gstm5 and Gstm6 [32]. These antioxidant genes are important in scavenging the ROS [83]. The upregulated genes imply that OPP confers a great ability to fight against oxidative stress in the heart, which is susceptible to prooxidant exposure [32]. The OPP antioxidant capacity is associated with free radical scavenging and hydrogen atom donation. The antioxidant capacities increase with respect to the phenolic compound hydroxylation degree [84,85]. Caffeoylshikimic acid, a major phenolic compound of OPP that contains four hydroxyl groups, might be responsible for the potent antioxidant activity. Moreover, other phenolic acids present in OPP including protocatechuic acid (PCA) and p-hydroxybenzoic acid (PHBA) have been anticipated to promote the antioxidant activity and when acting together, they may exert synergistic effects [23]. A previous study has revealed that antioxidant capacity of palm oil also depends on fruit maturation process, whereby the oil harvested during earlier ripening stages is suitable to be used as an ingredient for polyphenols-rich food and nutraceutical intention. This is because the phenolic content and antioxidant capacity may reduce with increased degree of fruit ripeness [86]. Therefore, it can be suggested that OPP should be extracted from palm oil fruit harvested at an earlier ripening stage to obtain the best functional antioxidant properties. 

In addition, an absorption characterization of phytochemicals extracted from palm fruit biomass has been performed in a recent study by bio-matching the plasma pharmacokinetic plasma peak (Tmax) with the onset of oxidative stress and inflammation (OSI). Through bio-matching of Tmax and OSI, the Phytochemical Absorption Prediction (PCAP) model and methodology were able to be predicted. From the study, it was shown that the post-consumption absorption of plasma peak (Tmax) ranged between 0.5 and 12 h and 2 and 6 h for intake in liquid and solid forms, respectively, and generally showed high antioxidant activity of the extracts. Thus, this might indicate that the phytochemicals of the palm fruit biomass have broad potential to be used for human health as dietary antioxidants in foods and nutraceutical products [87]. To date, the general pharmacokinetics of OPP have not yet been elucidated. However, the pharmacokinetics of OPP may be similar to carbohydrates, since on a dry weight basis, majority of the oil palm vegetation liquor (98%) are composed of fruit sugars, soluble fibers, organic acids and water-soluble vitamins. Meanwhile, the phenolic content in the oil palm vegetation liquor ranges from 1.38% to 2.43% [23]. 

### 3.3. Anti-Inflammatory Effects

Inflammatory events are crucial in the pathogenesis of CVD. The role of cytokines is progressively apparent as studies have proven the association of inflammatory/immune mechanisms in atherosclerosis and heart failure [88]. In the immune system, cytokines are responsible for serving as hormonal messengers in processes including cell-mediated immunity and allergic-type responses. Functionally, cytokines are categorized into two major groups namely proinflammatory and anti-inflammatory. Cytokines are mostly originated from T lymphocytes, which consist of two different molecules on the cell surface known as CD4 and CD8. T cells with CD4 cell surface molecules are recognized as helper T cells (Th). Th can be sub-categorized into Th1-type cytokines and Th2-type cytokines. The Th1-type cytokines include interferon-gamma (IFNγ) to generate pro-inflammatory reactions enacted for destroying intracellular parasites and preserving autoimmune reactions. Excessive pro-inflammatory reactions can contribute to abandoned tissue damage that should be countered. In contrast to the Th1-type cytokines, the Th2-type cytokines possess an anti-inflammatory property consisting IL-10, IL-4, IL-5 and IL-13. These cytokines are responsible for IgE promotion and eosinophilic reactions during allergic inflammation. With further anti-inflammatory Th2 reactions, the Th1-proinflammatory action can be counteracted. Therefore, an ideal scenario is that humans should have an equal Th1 and Th2 reactions to the immune system threat [89]. In the pathogenesis of atherosclerosis, Th1 is the dominant cytokine involved, which subsequently promotes the secretion of proinflammatory cytokines such as IFN-γ, IL-2, as well as TNF-α and -β [90]. The secretion of these cytokines may cause induced innate immune responses via stimulation of macrophages and vascular cells. During atheroma development, only a minor quantity of Th2 cytokines (IL-4, IL-5, and IL-10) are involved [90]. Moreover, there was a study that reported an association between Th2 immunity and reduction of CVD risk (myocardial infraction and stroke) [91].

Leow and colleagues demonstrated that the OPP has modulated Th1/Th2 axis of the immune system towards the Th2 cytokines that possess anti-inflammatory actions. This modulation might in turn contribute to the reduction of atherosclerosis development. Moreover, in comparison to the control mice, OPP supplementation to atherogenic-diet fed mice had significantly reduced pro-inflammatory IL-12 cytokine while significantly increased anti-inflammatory IL-13 cytokine [27]. The changes in cytokine levels may promote the anti-inflammatory response, thus preventing atherosclerosis development. IL-12, an innate immunity cytokine, has been implicated in atherosclerosis and other inflammatory diseases [92,93,94]. Activated macrophages and dendritic cells are responsible to secrete IL-12, which may induce cell-mediated immunity that consists of IFN-γ production and CD4+ Th1 cells differentiation. Additionally, IL-12 may enhance cytolytic functions by activating natural killer cells and CD8+ cytolytic T lymphocytes [93]. In contrast to IL-12, IL-13 is an adaptive immunity cytokine secreted by CD4+ Th2 cells. Generally, IL-13 acts by inhibiting the activity by macrophages and antagonizing IFN-γ, which is a crucial trigger for the formation and release of ROS [93]. 

### 3.4. Other Mechanisms: Fatty Acid Beta Oxidation, Tricarboxylic Acid (TCA) cycle and Electron Transport Chain (ETC)

Fatty acids are part of the lipid macronutrient class commonly found in the nature, food and organisms. They act as an important constituent of the cell membrane and possess significant biological, structural and functional roles. They have a vital role to generate a source of energy. Their metabolism yields a large quantity of adenosine triphosphate (ATP), in which the major pathway is mitochondrial fatty acid β-oxidation (FAO) [95,96]. This pathway is mostly used by organs such as the heart, liver and muscular tissues to obtain energy [96]. FAO also plays an important role when glucose supply becomes limited such as during fasting. Under limited glucose supply, majority of the tissues in the body except the brain should be able to directly utilize fatty acids to generate energy. In hepatic tissues, conversion of fatty acids into ketone bodies may occur to provide an additional energy source for all tissues including the brain [97]. 

A microarray data from the liver of BALB/c mice supplemented with OPP showed the upregulation of FAO genes, which indicated increased hepatic lipid catabolism [32]. These upregulated genes may suppress liver fat and visceral fat accumulation. Subsequently, the suppression of lipid accumulation through FAO may prevent lipid peroxidation leading to atherosclerosis [98]. This may eventually demonstrate that OPP is able to reduce atherosclerosis as a result of its ability in upregulating the hepatic FAO. The enhanced expression of hepatic lipid catabolism genes has also been reported in other polyphenolic compounds; catechins [99] and chlorogenic acids [100]. In a study by Murase et al. (2002), the supplementation with catechins in green tea showed a significant increase in mRNA expression of acyl-CoA oxidase and acyl-CoA dehydrogenase. Moreover, there was also significant beta-oxidation activity in the liver that may contribute to the reduction of CVD risk [99]. This implies that OPP may exert comparable effects to other dietary phenolic compounds affecting the FAO. 

Tricarboxylic Acid (TCA) cycle or Kreb’s cycle is the principal metabolic pathway that produces energy in cells. In order to produce the energy by means of adenosine triphosphate (ATP), oxidation of the reduced coenzymes by the electron transport chain (ETC) may takes place [101]. TCA releases stored energy through the oxidation of acetyl-coA derived from glycolysis, FAO and from the breakdown of ketone bodies and amino acids [102]. A high rate of ATP production and turnover is required by vital organs including the heart to sustain their mechanical work. Any disruption in the process of ATP generation may therefore affect contractile function directly [103]. Under normal conditions, most of the cardiac ATP (70% to 90%) is produced by the FAO. Meanwhile, the remaining cardiac ATP production (10% to 30%) comes from the oxidation of glucose, lactate, ketone bodies and certain amino acids [104]. Ageing process of the heart may alter the metabolism process towards carbohydrate metabolism (transcriptional shift from fatty acid metabolism) [103,105]. 

OPP has been demonstrated to prevent metabolic shift towards glycolysis by upregulating a key enzyme, Pdk4 [32]. Pdk4 inhibits pyruvate dehydrogenase activity, causing increased acetyl-coA influx from FAO into the TCA cycle. The acetyl-coA influx into TCA cycle may enhance FA oxidation and minimize carbohydrate metabolism by averting the glycolytic products into the TCA cycle [106]. This further suggests the OPP ability to upregulate Pdk4 and prevent metabolic alteration as well as ageing in the heart. 

Alterations in proteins have been identified to be associated with heart failure development. For example, the downregulation of uncoupling proteins (UCPs) may interfere with the regulation of mitochondrial membrane potential, energy metabolism and may promote ROS generation [107]. OPP can upregulate Ucp3 gene, which is then transcribed and translated to an uncoupling protein that provides protection against mitochondrial oxidative damage via reduction of ROS production [32]. Ucp3 is involved in fatty acids transporting out from the mitochondria and therefore is able to preserve the organelle from fatty acid anions or peroxides [108]. Other than that, OPP can also downregulate the genes involved in the heart TCA cycle including those genes encoding proteins which produce NADH (Mdh1) and FADH2 (Shdb) as well as those genes encoding subunits of succinate-CoA ligases which convert GTP/ATP to GDP/ADP (Suclg1 and Sucla2) [32]. 

Electron transport chain (ETC), which occurs in the inner membrane of mitochondria is important for oxidative metabolism. ETC consists of several substantial protein complexes (Complex I–V). While electrons flow through the ETC, active proton pumping occurs across the membrane which subsequently creates an electrical potential and pH gradient across the membrane. The free energies of this pump may cumulatively contain proton motive force. The discharge of force through the final complex of the chain, complex V, triggers the conversion of ADP and Pi to ATP [109]. Leow et al. (2011) found that OPP had downregulated several genes that encode proteins in Complex I, II and V [32]. These results indicate that OPP has downregulated the energy production in the heart. Therefore, through the reduction of energy production, OPP may play a role as antioxidant to reduce the presence of ROS in the heart, thus suggesting a significant positive effect of OPP in preventing cardiac oxidative stress.

## 4. Safety and Toxicity of OPP

In a previous study, OPP provided at antioxidant value of 1500 ppm GAE as ad libitum drinking fluids to mice showed no toxicity effects, as indicated by insignificant alteration to the organ histology, hematology and clinical biochemistry analyses. OPP had upregulated three genes namely Cxcl12, Gjb1 and Ar [32]. These genes were downregulated during the administration of comfrey, a herbal plant containing pyrrolizidine alkaloids, which was associated with hepatoxicity in humans [110]. Thus, the upregulation of these three genes by OPP might imply that OPP may not trigger hepatoxicity. 

**Figure 3:**
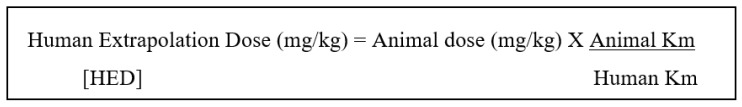
Formula for dose translation from animal to human based on Body Surface Area (BSA). Source: [113].

Sambanthamurthi and colleagues performed a teratological study by monitoring three generations of Sprague Dawley rats supplemented with 1500 or 2400 mg/L GAE OPP as the only drinking fluid for 21–22 days (vaginal plug identification until the exact delivery day). For both doses of OPP, the offspring of rats showed the absence of developmental birth defects or congenital anomalies. Moreover, the OPP supplementation to the rats did not affect their well-being. The macroscopic observation on all major organs showed no signs of OPP-induced toxicity [23]. Several indications from the studied animals also revealed that OPP supplementation up to 3000 mg GAE/L dosage was safe, as shown by the histology, hematology and clinical biochemistry aspects [27,111,112]. Extrapolating animal dosage to humans for phase I and phase II clinical trials should be performed by appropriate conversion of drug doses such as body surface area (BSA) normalization method, as shown below (Figure 3) [113]. 

The safety aspects of OPP administration is not limited only to pre-clinical data, as it has been evaluated in phase I single-blind trial in healthy volunteers. Following the daily administration of 450 mg GAE/day OPP for 60 days, there was no major or serious adverse effect detected. Normal range of clinical biochemistry profiles implied that OPP supplementation was not toxic. None of them reported any hypersensitivity reactions throughout the treatment period [34]. Thus, OPP supplementation at the specific dose is generally safe to be consumed. 

## 5. Conclusions

This review summarized the current evidence of OPP for cardiovascular health. In the future, studies from in vitro systems should be well-integrated with in vivo studies, as well as clinical trials to be conducted to evaluate the applicability of these proposed mechanisms of OPP in human cardiovascular health. Therefore, in elucidating the OPP safety profile and potential as an alternative cardioprotective agent, further in-depth studies are warranted to explore a conclusive mechanism for its therapeutic action. Currently, there are several mechanisms proposed for its cardioprotective effects. The understanding of the proposed mechanism that leads to cardiovascular health will provide more insight into the development of potent cardioprotective agents that specifically target the pathways. The proposed pathways include the downregulated genes of cholesterol biosynthesis pathway that could mimic the action of a well-known anti-hyperlipidemic agent, for example, statin, by targeting the HMG-CoA reductase. Other mechanisms involved are the antioxidative and anti-inflammatory properties as well as modulation of vital clinical biochemistry pathway including fatty acid β-oxidation, TCA and ETC. In conclusion, the health benefits of OPP for CVD have been clearly elucidated by literature and it is important to conserve this valuable nutraceutical derived from the oil palm fruit. 

## Figures and Tables

**Table 1:** The effects of oil palm phenolics (OPP) in cardiovascular health.

Study Type	Sample/Population	Intervention Dose and Route	Findings	Reference
Animal study	Male New Zealand white rabbits induced with an atherogenic diet for 100 days.	OPP (1500 mg GAE/L) in drinking fluid.	OPP treatment showed no significant difference in plasma lipid profile, while slightly higher in the high-density lipoprotein cholesterol (HDL-C) level compared to control. OPP treatment resulted in significantly lower fatty streaks development compared to the control (*p* < 0.05). OPP treatment indicated proliferation of smooth muscle, development of intimal fibrosis and extracellular lipid. However, lipid core or necrosis was absent. The formation of foam cell was at lesser degree.	[28]
Animal study	Male spontaneously hypertensive rats (SHR).	OPP at 1500 and 3000 GAE for 20 weeks, as a beverage (30 mL/rat/day).	The prolonged OPP supplementation at 1500 mg/L GAE did not affect blood pressure in this model.	[33]
Animal study	Sprague-Dawley rats induced hypertension with L-NAME.	OPP at 1500 mg/L and 3000 mg/L GAE) as a beverage (30 mL/rat/day) for 4 weeks prior to L-NAME induction. Treatments were continued for a further two months thereafter.	OPP treatment at 3000 mg/L GAE significantly lowered the blood pressure in the L-NAME hypertension model (*p* < 0.001) compared to OPP dosed at 1500 mg/L GAE (*p* < 0.01). OPP treatment at both doses did not return BP to a complete normalization to the baseline level of the control group. OPP treatment at 3000 mg/L GAE significantly reduced the duration of ventricular tachycardia (*p* < 0.01). There were no episodes of ventricular fibrillation occurred when compared to the control group.	
Animal study	Male SHR.	Fractionated OPP (as a single oral dose, 250 mg/kg) was introduced via pipette positioned at the back of the tongue of SHR.	The OPP fractions reduced mean BP (7–27 mmHg) within 6 h post-administration. Both OPP fractions showed the highest BP reduction at 6 h post-administration.	
Animal study	Male Wistar Kyoto rats fed with pro-arrhythmic diet.	OPP (1500 mg/L GAE) was given as beverage (30 mL/rat/day).	OPP significantly reduced the ventricular fibrillation (VF) incidences when compared to the control group (*p* < 0.05). OPP-treated group had a lower percentage of VF (52%) compared to the control group (90%) OPP-treated group had lower mortality (20%) compared to control rats (40%) There was no difference in ischemic-affected myocardium area (zone-at-risk) between OPP and control groups.	
Ex vivo	Isolated segments (3 mm) of the thoracic aorta and mesenteric arterial bed from male normotensive Wistar Kyoto rats and SHR.	OPP was introduced at the following doses: 0.25, 0.50, and 1.00 mg/kg to pre-contracted vascular preparations in the organ bath chamber.	In a dose-dependently manner, OPP enhanced vascular relaxation in both ex vivo systems; isolated aortic rings (conductance vessels) and perfused mesenteric vascular bed (resistance vessels).	[23]
In vitro	Conjugated dienes.	OPP extracts were added to low-density lipoprotein cholesterols (LDL-C) immediately before the addition of oxidant (copper sulphate).	In a dose-dependently manner, OPP prevented the Cu-mediated LDL oxidation. OPP have delayed the duration of conjugated diene formation when compared to the control.	
Animal study	Male inbred BALB/c mice.	OPP in drinking fluids ad libitum 1500 GAE mg/L.	OPP have upregulated four lipid catabolism genes (Acadl, Acads, Hadhb, Hadhsc) and downregulated five cholesterol biosynthesis genes (Hmgcs1, Lss, Sc4mol, Fdps, Nsdhl)	[32]
Animal study	Male inbred BALB/c mice.	OPP in drinking fluids ad libitum at 1500 ppm GAE mg/L.	OPP-treated group have significantly increased the total cholersterol (TC), LDL and HDL levels OPP have downregulated the genes expressed in the presentation of endogenous antigen, metabolism of fatty acids, enzymatic activities of NADH dehydrogenase (ubiquinone) and oxidoreductase. OPP have upregulated genes expressed in the heart antioxidant activity; Gpx1 and Mgst1	[27]
Human study	25 volunteers Normolipidemic, nonsmokers, and no clinical symptoms associated with CVD.	OPP was supplemented as 300 mL beverage (containing 450 mg/GAE/day).	Following the 60 days OPP supplementation, plasma TC and LDL-C levels were significantly lower compared to the control treatment, with *p* = 0.025 and *p* = 0.04, respectively. However, the OPP-supplemented group showed insignificant changes in HDL-C, triacylglycerol (TAG) and TC/HDL ratio when compared to control treatments.	[34]

## Abbreviations

Acadl	acetyl-CoA dehydrogenase
Acads	acyl-CoA dehydrogenase
Ar	androgen receptor
Cxcl12	chemokine (C-X-C motif) ligand 12
Fdps	farnesyl diphosphate synthase
Gjb1	gap junction membrane channel protein beta 1
Gstm2, Gstm5, Gstm6	glutathione S transferases
Gpx1	glutathione peroxidase 1
Hadhb, Hadhsc	hydroxyacyl-CoA dehydrogenases
Hmgcs1	3-hydroxy-3-methylglutaryl-CoA synthase 1
Lss	lanosterol synthase
Mgst1	microsomal glutathione S-transferase 1
Nsdhl	NAD(P) dependent steroid dehydrogenase-like
Sc4mol	sterol-C4-methyl oxidase-like
